# SNAP food purchase restrictions and retailer availability: a difference-in-differences analysis

**DOI:** 10.1093/haschl/qxag275

**Published:** 2026-10-10

**Authors:** Benjamin W Chrisinger, Justin S White

**Affiliations:** Department of Community Health, Tufts University, Medford, MA 02155, United States; Center for Health and Wellbeing, Princeton University, Princeton, NJ 08544, United States; Department of Health Law, Policy & Management, Boston University School of Public Health, Boston, MA 02118, United States

**Keywords:** supplemental nutrition assistance program, state policy, food access, difference-in-differences

## Abstract

**Introduction:**

States have begun implementing waivers that prohibit the Supplemental Nutrition Assistance Program (SNAP) benefits from being used to purchase selected foods and beverages. We assessed whether these restrictions were associated with changes in the availability of SNAP-authorized convenience stores, supermarkets, and superstores.

**Methods:**

We analyzed repeated observations from 83 697 Census tracts from January 2025 through June 2026. Difference-in-differences models compared changes in retailer availability in states implementing waivers with concurrent changes in states without implemented waivers.

**Results:**

Waiver implementation was associated with a 1.4% decline in SNAP-authorized convenience stores, equivalent to 3.0 fewer stores per 100 tracts (95% CI, −5.7 to −0.4; *P* = 0.03), but not with a significant change in supermarkets or superstores. The largest declines in convenience stores occurred among states restricting beverages and candy (−2.2%; −4.9 stores per 100 tracts; 95% CI, −6.9 to −2.9; *P* < 0.01).

**Conclusion:**

The SNAP food restrictions were associated with reduced availability of SNAP-authorized convenience stores but not supermarkets or superstores. Continued monitoring is warranted as these policies expand.

Key Points

- The Supplemental Nutrition Assistance Program (SNAP) waiver implementation was associated with a 1.4% decline in SNAP-authorized convenience stores, equivalent to 3.0 fewer stores per 100 tracts, but no significant change in supermarkets or superstores.
- The largest declines in SNAP-authorized convenience stores were observed in states restricting beverages and candy, although this pattern was partly influenced by Texas.
- These early findings suggest that SNAP food purchase restrictions may reduce the availability of SNAP-authorized convenience stores, underscoring the need to monitor retailer participation as these policies are implemented.

## Introduction

The Supplemental Nutrition Assistance Program (SNAP) is the largest federal nutrition program and the nation's primary guard against food insecurity. Since January 2026, 10 states have implemented waivers granted by the US Department of Agriculture (USDA) that restrict certain foods and beverages purchasable with SNAP benefits. Although definitions vary, all implemented waivers restrict some sugar-sweetened beverages, and some also restrict candy or other foods.^1^

More than 450 000 retailers are authorized to accept SNAP benefits.^2^ Retailer participation is voluntary. New food restrictions may increase retailers' administrative burden by requiring retailers to modify checkout systems, train staff, and customers and prevent purchases of restricted products. Retailers that repeatedly allow prohibited purchases may face removal from SNAP.

These new requirements have raised concerns that some retailers, particularly stores with limited administrative capacity, may stop participating in SNAP, potentially reducing food access.^3^ We examined whether implementation of state SNAP food purchase restrictions was associated with changes in the availability of SNAP-authorized convenience stores, supermarkets, and superstores.

## Data and methods

### Data sources and variable generation

Information about food restriction waivers was obtained from USDA postings, including waiver submission, approval, and implementation dates and restricted product categories.^4^ We classified the 10 states implementing restrictions during the study period as restricting beverages only (Nebraska, Utah, West Virginia), beverages and candy (Indiana, Idaho, Oklahoma, Louisiana, Texas), or beverages, candy, and additional foods (Iowa, Florida).

USDA updates a public database of SNAP-authorized retailers twice monthly, including retailer geocoordinates and store-type classifications.^2^ We obtained archived files from January 1, 2025 through June 23, 2026, yielding 33 observation periods. Retailer locations were assigned to 2024 Census tracts in the 50 states and District of Columbia, producing a tract-period panel of 2 762 001 observations across 83 697 tracts. Outcomes were counts of SNAP-authorized convenience stores and supermarkets or superstores per tract-period.

American Community Survey 2020-2024 5-year estimates were used to measure tract population, population density, median household income, and SNAP household participation. Tracts were assigned to state-specific quintiles of income, population density, and SNAP participation (Appendix Tables AS1-AS3). We also identified tracts within 10 miles of a state border and distinguished those adjacent to states with active waivers. To account for concurrent SNAP policy changes following the July 2025 federal spending bill (H.R.1),^5,6^ models included an interaction between the post-H.R.1 period and standardized pre-H.R.1 tract SNAP participation.

### Statistical analysis

#### Primary analysis

We used difference-in-differences models, which compared before-to-after changes in SNAP retailer availability in waiver-implementing states with concurrent changes in states that had not implemented waivers. Models included separate indicators for the period after waiver submission but before implementation and after waiver implementation, and for the period after implementation, allowing us to assess whether retailer availability began changing before restrictions took effect. We also tested whether the post-implementation estimate differed from the post-submission/pre-implementation estimate.

Models included state and biweekly-period fixed effects and adjusted for log population density, SNAP household share, White population share, median household income, and an interaction between the period following the July 2025 federal SNAP policy changes under H.R.1 and baseline SNAP participation. Standard errors were clustered by state. Models were restricted to populated tracts and, for each outcome, tracts with at least one store of that type before the first waiver submission observed in the sample. Estimates are reported as changes in stores per tract and percentage changes relative to the treated pre-implementation mean.

Adjusted event-study models replaced the implementation indicator with indicators for biweekly periods relative to implementation, with the period immediately before implementation as the reference, while retaining the fixed effects and covariates from the primary specification.

#### Sensitivity analyses

Sensitivity analyses used population-weighting, Callaway-Sant’Anna difference-in-differences models for policies implemented at different times,^7^ leave-one-treated-state-out analyses, and adjustment for monthly state SNAP participation. We also used wild-cluster-bootstrap inference, a resampling method intended to improve statistical inference when policy changes occur in relatively few states. Additional results are reported in the Appendix.

## Results

Overall, 19 304 tracts were in states with implemented SNAP waivers; 13 536 treated-state tracts had at least one SNAP-authorized convenience store and 6490 had at least one supermarket or superstore in the analytic sample (Appendix Table AS4).

In adjusted difference-in-differences models, waiver implementation was associated with a 1.4% decline in SNAP-authorized convenience stores, equivalent to 3.0 fewer stores per 100 tracts (95% CI, −5.7 to −0.4; *P* = 0.03), but was not associated with a significant change in supermarkets/superstores (−0.3%; −0.4 stores per 100 tracts; 95% CI, −1.6 to 0.8; *P* = 0.52). The largest convenience-store decline occurred in states restricting beverages and candy (−2.2%; −4.9 stores per 100 tracts; 95% CI, −6.9 to −2.9; *P* < 0.01); estimates were smaller and not statistically significant for beverage-only restrictions or restrictions covering beverages, candy, and additional foods. Supermarket and superstore estimates were small and not statistically significant across waiver types (Figure 1).

**Figure 1.**
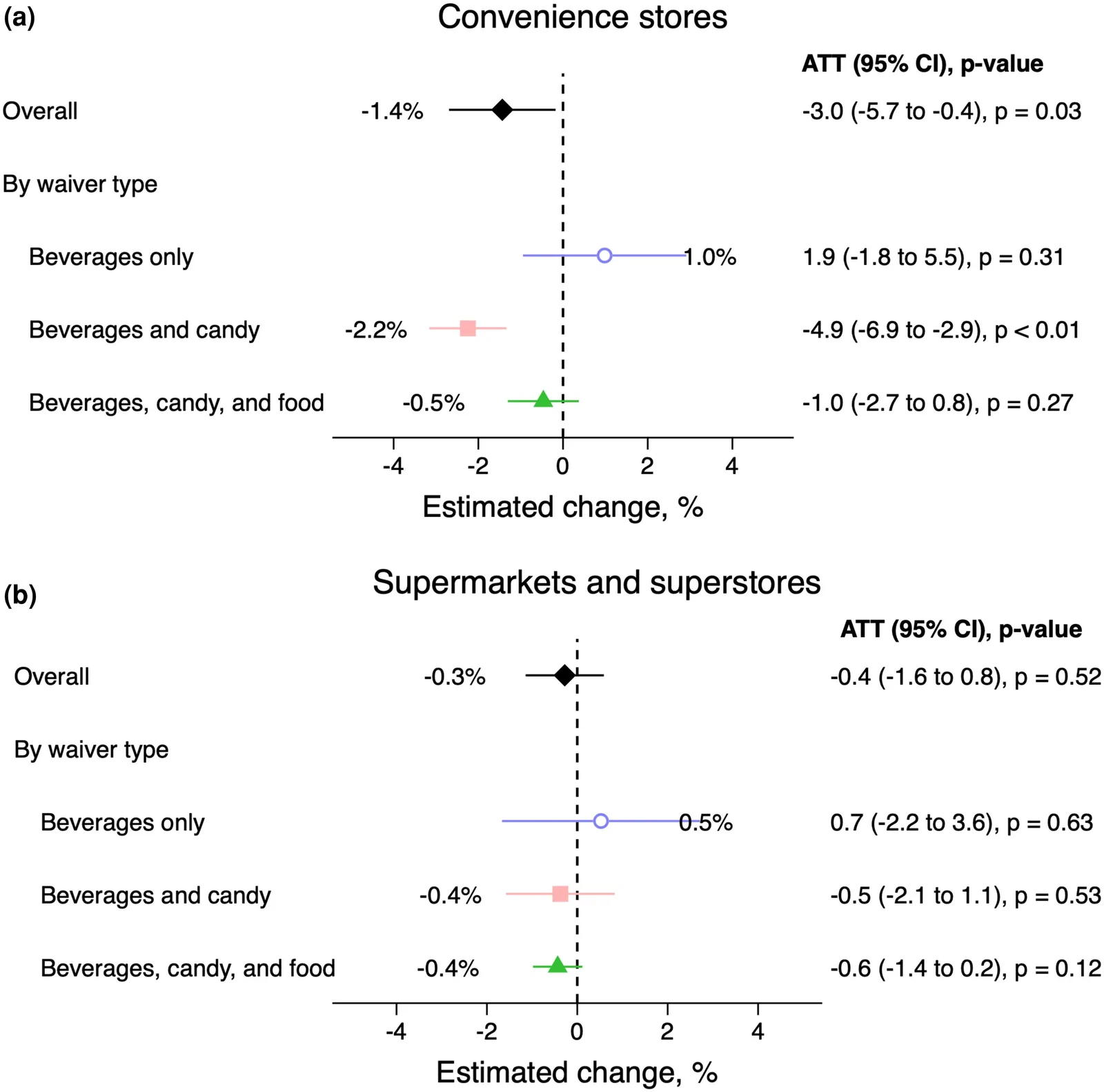
Estimated changes in food retail availability after SNAP waiver implementation, overall and by waiver type. Adjusted two-way fixed-effects difference-in-differences estimates overall and by waiver type for convenience stores (a) and supermarkets/superstores (b). Points indicate percentage changes relative to treated-state pre-implementation means; lines indicate 95% CIs with standard errors clustered by state. Right-hand labels report changes per 100 tracts and two-sided *P*-values. Models included tracts with at least one relevant store before the first observed waiver submission observed in the sample. SNAP, Supplemental Nutrition Assistance Program.

There was little evidence that convenience-store availability changed after waiver submission but before implementation (−0.3%; *P* = 0.45). After implementation, the estimate was an additional 1.1% lower than during the post-submission/pre-implementation period (*P* = 0.004). Event-study estimates likewise showed no clear systematic divergence before implementation and became negative after implementation, while supermarket and superstore estimates remained imprecise (Figure 2). In subgroup analyses, convenience store declines were observed in several quintiles of lower- and middle-income quintiles and in the highest quintiles of baseline SNAP participation (Appendix Figures AS1 and AS2). The difference between border and non-border tracts was not statistically significant. Population-weighted and Callaway-Sant’Anna difference-in-differences estimates were similar (Appendix Figures AS3-AS7).

**Figure 2.**
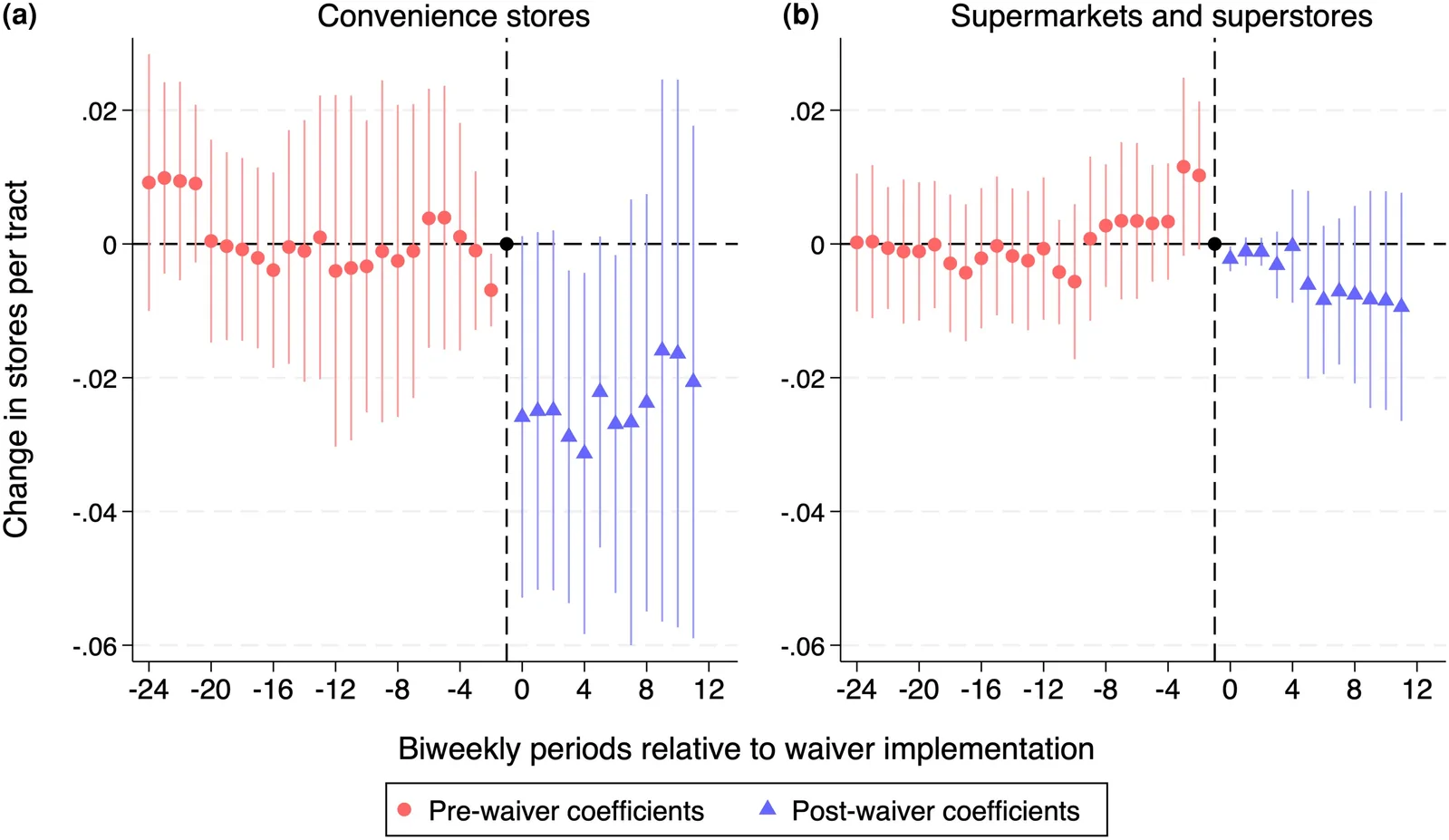
Event study estimates of changes in food retail availability after SNAP waiver implementation. Adjusted two-way fixed-effects estimates for convenience stores (a) and supermarkets/superstores (b), relative to the biweekly period before implementation. Points show coefficients and lines 95% CIs with standard errors clustered by state. Colors and shapes distinguish pre-implementation, post-implementation, and reference periods. Models used the primary analytic sample and specification. SNAP, Supplemental Nutrition Assistance Program.

Leave-one-treated-state-out analyses indicated that Texas was influential: excluding Texas attenuated the convenience-store estimate from −1.4% to −0.7%, with the confidence interval including zero, whereas excluding other treated states produced estimates closer to the full-sample result (Appendix Figure AS8). The Supplemental Nutrition Assistance Program participation declined somewhat more in waiver states than in comparison states after July 2025, although the differential changes were not statistically significant for either households (−2.1%; *P* = 0.15) or persons (−2.1%; *P* = 0.17) (Appendix Figure AS9; Appendix Table AS5). Among months with contemporaneous caseload data, adjustment for SNAP households changed the convenience-store estimate minimally, from −1.21% to −1.22%. Wild-cluster-bootstrap inference produced a wider confidence interval for the primary convenience-store estimate (95% CI, −5.8 to 0.3 stores per 100 tracts; *P* = 0.075) (Appendix Table AS6).

## Discussion

Implementation of SNAP food restriction waivers was associated with fewer SNAP-authorized convenience stores, but not supermarkets and superstores. Event-study estimates were consistent with a decline in convenience stores after implementation rather than after waiver submission. Larger retailers may have greater administrative and technical capacity to modify checkout systems, train staff, and monitor compliance, whereas smaller retailers may face higher relative costs of remaining SNAP-authorized under new product restrictions.

The largest declines occurred among states restricting beverages and candy, although this pattern should be interpreted cautiously. Texas, which implemented this type of waiver and accounted for a substantial share of treated tracts, materially influenced the pooled estimate. Excluding Texas reduced the estimated convenience-store decline by about half. Subgroup estimates also suggested larger declines in several lower- and middle-income tracts and higher SNAP participation tracts. Non-border tracts also showed larger declines than border tracts not adjacent to active waiver states, although the formal interaction was not statistically significant. Border area retail markets may also differ from interior markets in ways not captured by observed tract characteristics. More generally, variation across states suggests that retailer responses may depend on features of implementation and local retail markets, not simply on the categories of foods restricted.

This study expands on prior work examining the characteristics of approved waivers by assessing outcomes related to the food retail environment.^1^ Other work has also documented changes in the SNAP retail environment over time (1997-2020),^8^ though to our knowledge, this is the first examination of changes in response to current SNAP waiver policies.

### Limitations

This study has several limitations. USDA files identify authorization status but not whether changes reflect store closure, voluntary withdrawal, disqualification, or administrative updating, and they do not distinguish chain from independent stores. Follow-up was short, and in some states ended before USDA's 90-day compliance grace period expired. Only 10 states implemented waivers, limiting precision and our ability to separate effects of waiver design from differences across states and implementation timing. Consistent with this concern, wild-cluster-bootstrap inference produced a wider confidence interval that included zero. Concurrent federal SNAP policy changes may also have affected retailer demand. Adjustment for contemporaneous SNAP participation did not materially change the estimates, although participation itself may respond to policy changes and these models were therefore treated as sensitivity analyses. A federal district court order on June 22, 2026 affecting implementation of several state waivers coincided with the final observation period and was therefore unlikely to affect the estimates.^9^

## Conclusion

These early findings suggest that SNAP food purchase restrictions may reduce the availability of SNAP-authorized convenience stores in some settings. Although retailer availability does not wholly determine diet,^10^ it is an important indicator of food access and equity.^11^ Convenience stores are not typically primary grocery destinations for SNAP participants;^12^ still, they may be important between larger shopping trips, particularly when larger stores are less accessible.^13^ Continued monitoring across additional states and longer follow-up will be important for understanding implications for retailer participation and food access.

## Supplementary Material

qxag275_Supplementary_Data

## Data Availability

Historical data on authorized SNAP retailers are publicly available at: https://www.fna.usda.gov/snap/retailer-locator/data. Data on currently-authorized stores are periodically posted to: https://www.fna.usda.gov/snap/retailer-locator.

## References

[1] Chrisinger BW . Characteristics of state waivers to establish nutritional restrictions in the supplemental nutrition assistance program. Health Aff Sch. 2025;3(12):qxaf221. 10.1093/haschl/qxaf221 PMC12684384 41368218

[2] USDA Food and Nutrition Service . SNAP retailer locator. Updated February 17, 2026. Accessed August 3, 2026. https://www.fna.usda.gov/snap/retailer-locator

[3] SNAP Food Restriction Waivers . Food Research & Action Center. Accessed July 7, 2026. https://frac.org/snap-restrictions

[4] USDA Food and Nutrition Service . SNAP food restriction waivers. Updated August 6, 2025. Accessed October 5, 2025. https://www.fna.usda.gov/snap/waivers/foodrestriction

[5] Rosenbaum D, Llobrera J, Nchako C, Nunez L. SNAP tracker: people are losing food assistance as the Republican megabill is implemented. Updated June 22, 2026. Accessed July 8, 2026. https://www.cbpp.org/research/food-assistance/snap-tracker-people-are-losing-food-assistance-as-the-republican-megabill

[6] Bleich SN, Plata-Nino G. Changes to SNAP under HR 1 and the implications for food insecurity. JAMA Health Forum. 2026;7(1):e260158. 10.1001/jamahealthforum.2026.0158 41609792

[7] Callaway B, Sant’Anna PHC. Difference-in-differences with multiple time periods. J Econom. 2021;225(2):200–230. 10.1016/j.jeconom.2020.12.001

[8] Chrisinger BW, Feng W. Characterizing the dynamic nature of SNAP food environments in the United States. AAEA Conference Paper. 2025. 10.22004/AG.ECON.360877

[9] Brittain B . US judge blocks Trump administration SNAP restrictions on soda, candy. *Reuters*. Updated June 22, 2026. Accessed July 7, 2026. https://www.reuters.com/legal/government/us-judge-blocks-trump-administration-snap-restrictions-soda-candy-2026-06-22/

[10] Allcott H, Diamond R, Dubé J-P, Handbury J, Rahkovsky I, Schnell M. Food deserts and the causes of nutritional inequality*. Q J Econ. 2019;134(4):1793–1844. 10.1093/qje/qjz015

[11] Li Q, Zhao S. Access to SNAP-authorized retailers and diet quality among SNAP recipients. JAMA Health Forum. 2025;6(4):e250677. 10.1001/jamahealthforum.2025.0677 PMC12008766 40249607

[12] Chrisinger BW, Kallan MJ, Whiteman ED, Hillier A. Where do U.S. households purchase healthy foods? An analysis of food-at-home purchases across different types of retailers in a nationally representative dataset. Prev Med. 2018;112:15–22. 10.1016/j.ypmed.2018.03.015 PMC5970989 29555187

[13] DiSantis KI, Hillier A, Holaday R, Kumanyika S. Why do you shop there? A mixed methods study mapping household food shopping patterns onto weekly routines of black women. Int J Behav Nutr Phys Act. 2016;13(1):11. 10.1186/s12966-016-0333-6 PMC4730755 26821926

