# Supplementary material for: SNAP food purchase restrictions and retailer availability: a difference-in-differences analysis
Source: Health Aff Sch. 2026 Oct 10;4(10):qxag275. doi: 10.1093/haschl/qxag275 (PMC13711658; doi:10.1093/haschl/qxag275)
Supplement: Supplementary file 1 [file qxag275_supplementary_data.zip › qxag275_Supplementary_Data.docx]

**Supplementary Appendix**

Supplement to: “SNAP Food Purchase Restrictions and Retailer Availability: A Difference-in-Differences Analysis”

**List of Appendix Figures**

[**Appendix Figure A1.** Estimated changes in convenience store availability after SNAP waiver implementation, overall and by tract subgroup	2](#_Toc239946112)

[**Appendix Figure A2.** Estimated changes in supermarket and superstore availability after SNAP waiver implementation, overall and by tract subgroup	3](#_Toc239946113)

[**Appendix Figure A3.** Estimated changes in food retail availability after SNAP waiver implementation, overall and by waiver type (population-weighted)	4](#_Toc239946114)

[**Appendix Figure A4.** Estimated changes in food retail availability after SNAP waiver implementation, overall and by tract subgroup (population-weighted)	5](#_Toc239946115)

[**Appendix Figure A5.** Callaway-Sant’Anna difference-in-differences estimates of the changes in food retail availability after SNAP waiver implementation, overall and by tract subgroup	7](#_Toc239946116)

[**Appendix Figure A6.** Callaway-Sant’Anna difference-in-differences estimates of the changes in food retail availability after SNAP waiver implementation, by implementation timing group	9](#_Toc239946117)

[**Appendix Figure A7.** Callaway-Sant’Anna difference-in-differences event study estimates of changes in food retail availability after SNAP waiver implementation	10](#_Toc239946118)

[**Appendix Figure A8.** Leave-one-treated-state-out estimates of changes in SNAP-authorized retailer availability after waiver implementation	11](#_Toc239946119)

[**Appendix Figure A9.** Trends in SNAP participation in waiver and comparison states	12](#_Toc239946120)

**List of Appendix Tables**

[**Appendix Table A1.** State Quintile Boundaries for Population Density (Persons per Square Mile; Min, Max)	13](#_Toc239946106)

[**Appendix Table A2.** State Quintile Boundaries for Median Household Income (Min, Max)	14](#_Toc239946107)

[**Appendix Table A3.** State Quintile Boundaries for Percent of Households Receiving SNAP (Min, Max)	15](#_Toc239946108)

[**Appendix Table A4.** Waiver characteristics and analytic sample by state	16](#_Toc239946109)

[**Appendix Table A5.** SNAP caseload trends and sensitivity of retailer estimates to contemporaneous SNAP participation	18](#_Toc239946111)

[**Appendix Table A6.** Conventional and wild-cluster-bootstrap inference for primary waiver implementation estimates	17](#_Toc239946110)

**Appendix Figure A1.** Estimated changes in convenience store availability after SNAP waiver implementation, overall and by tract subgroup

Note: Estimates are from adjusted two-way fixed-effects difference-in-differences models. Points indicate estimated average treatment effects on the treated (ATTs); horizontal lines indicate 95% CIs. The x-axis and labels adjacent to the points and whiskers express the ATT as a percentage change relative to the pre-implementation mean for the corresponding subgroup. Labels on the secondary y-axis express the ATT as the change in stores per 100 census tracts, with 95% CIs and p-values. Models are restricted to census tracts with at least one store of the relevant type in any biweekly period before the first waiver submission observed in the sample. The Border subgroup includes tracts within 10 miles of a state border but excludes border tracts adjacent to a state with an active waiver.

**Appendix Figure A2.** Estimated changes in supermarket and superstore availability after SNAP waiver implementation, overall and by tract subgroup

Note: Estimates are from adjusted two-way fixed-effects difference-in-differences models. Points indicate estimated average treatment effects on the treated (ATTs); horizontal lines indicate 95% CIs. The x-axis and labels adjacent to the points and whiskers express the ATT as a percentage change relative to the pre-implementation mean for the corresponding subgroup. Labels on the secondary y-axis express the ATT as the change in stores per 100 census tracts, with 95% CIs and p-values. Models are restricted to census tracts with at least one store of the relevant type in any biweekly period before the first waiver submission observed in the sample. The Border subgroup includes tracts within 10 miles of a state border but excludes border tracts adjacent to a state with an active waiver.

**Appendix Figure A3.** Estimated changes in food retail availability after SNAP waiver implementation, overall and by waiver type (population-weighted)

Note: Estimates are from population-weighted, adjusted two-way fixed-effects difference-in-differences models. Points indicate estimated average treatment effects on the treated (ATTs); horizontal lines indicate 95% CIs. The x-axis and labels adjacent to the points and whiskers express the ATT as a percentage change relative to the pre-implementation mean for the corresponding waiver type. Labels on the secondary y-axis express the ATT as the change in stores per 100 census tracts, with 95% CIs and P values. Waiver types are categorized according to whether the implemented restrictions applied to sugar-sweetened beverages only; sugar-sweetened beverages and candy; or sugar-sweetened beverages, candy, and additional foods. Models are restricted to census tracts with at least one store of the relevant type in any biweekly period before the first waiver submission observed in the sample. Panel A shows convenience stores; Panel B shows supermarkets and superstores.

**Appendix Figure A4.** Estimated changes in food retail availability after SNAP waiver implementation, overall and by tract subgroup (population-weighted)

Note: Estimates are from population-weighted adjusted two-way fixed-effects difference-in-differences models. Points indicate estimated average treatment effects on the treated (ATTs); horizontal lines indicate 95% CIs. The x-axis and labels adjacent to the points and whiskers express the ATT as a percentage change relative to the pre-implementation mean for the corresponding subgroup. Labels on the secondary y-axis express the ATT as the change in stores per 100 census tracts, with 95% CIs and p-values. Models are restricted to census tracts with at least one store of the relevant type in any biweekly period before the first waiver submission observed in the sample. The Border subgroup includes tracts within 10 miles of a state border but excludes border tracts adjacent to a state with an active waiver. Panel A shows convenience stores; Panel B shows supermarkets and superstores.

**Appendix Figure A5.** Callaway-Sant’Anna difference-in-differences estimates of the changes in food retail availability after SNAP waiver implementation, overall and by tract subgroup

Note: Estimates are from Callaway and Sant’Anna difference-in-differences models for staggered policy implementation. Points indicate estimated average treatment effects on the treated (ATTs); horizontal lines indicate 95% CIs. The x-axis and labels adjacent to the points and whiskers express the ATT as a percentage change relative to the pre-implementation mean for the corresponding subgroup. Labels on the secondary y-axis express the ATT as the change in stores per 100 census tracts, with 95% CIs and p-values. Models are restricted to census tracts with at least one store of the relevant type in any biweekly period before the first waiver submission observed in the sample. The Border subgroup includes tracts within 10 miles of a state border but excludes border tracts adjacent to a state with an active waiver. Panel A shows convenience stores; Panel B shows supermarkets and superstores.

**Appendix Figure A6.** Callaway-Sant’Anna difference-in-differences estimates of the changes in food retail availability after SNAP waiver implementation, by implementation timing group

Note: Estimates are from Callaway and Sant’Anna difference-in-differences models for staggered policy implementation. Points indicate estimated average treatment effects on the treated (ATTs); horizontal lines indicate 95% CIs. The y-axis expresses the ATT as the change in stores per census tract. Implementation timing groups are defined according to the first biweekly period in which the SNAP waiver was implemented; the “Average” estimate represents the overall ATT aggregated across implementation timing groups. Models are restricted to census tracts with at least one store of the relevant type in any biweekly period before the first waiver submission observed in the sample. Panel A shows convenience stores; Panel B shows supermarkets and superstores.

**Appendix Figure A7.** Callaway-Sant’Anna difference-in-differences event study estimates of changes in food retail availability after SNAP waiver implementation

Note: Estimates are from Callaway and Sant’Anna difference-in-differences models for staggered policy implementation. Points indicate event-time coefficients; vertical lines indicate 95% CIs. Coefficients are expressed as changes in stores per census tract relative to the reference period, defined as the biweekly period immediately before waiver implementation. Red circles indicate pre-implementation coefficients, blue triangles indicate postimplementation coefficients, and the black point denotes the omitted reference period. Models are restricted to census tracts with at least one store of the relevant type in any biweekly period before the first waiver submission observed in the sample. Panel A shows convenience stores; Panel B shows supermarkets and superstores.

**Appendix Figure A8.** Leave-one-treated-state-out estimates of changes in SNAP-authorized retailer availability after waiver implementation

Note: Estimates are from the primary adjusted two-way fixed-effects difference-in-differences model, sequentially omitting each waiver-implementing state. Points indicate estimated changes in SNAP-authorized stores per 100 Census tracts; horizontal lines indicate 95% confidence intervals with standard errors clustered by state. The diamond shows the full-sample estimate.

**Appendix Figure A9.** Trends in SNAP participation in waiver and comparison states

Note: Panel A shows SNAP-participating households and Panel B shows SNAP-participating persons. For each state, monthly participation is expressed as the percentage change from its January-March 2025 mean; plotted values are unweighted means across states. The vertical line denotes enactment of H.R.1 in July 2025.

**Appendix Table A1.** State Quintile Boundaries for Population Density (Persons per Square Mile; Min, Max)

| State | Q1 | Q2 | Q3 | Q4 | Q5 |
| --- | --- | --- | --- | --- | --- |
| AK | 0, 1.01 | 1.02, 55.96 | 65.64, 719.06 | 721.97, 3078.76 | 3461.39, 11415.94 |
| AL | 0, 56.04 | 56.4, 195.47 | 195.5, 677.09 | 684.13, 1863.57 | 1864.99, 13355.78 |
| AR | 0, 24.54 | 24.55, 111.36 | 112.07, 456.82 | 458.48, 1675.1 | 1708.94, 11863.29 |
| AZ | 0, 465.95 | 472.64, 2237.83 | 2241.73, 4155.51 | 4160.63, 5993.05 | 5993.65, 34337.01 |
| CA | 0, 1848.64 | 1848.92, 4974.6 | 4974.89, 7888.97 | 7889.32, 12416.62 | 12428.26, 190925.84 |
| CO | 0, 194.38 | 197.9, 2013.65 | 2015.45, 4035.61 | 4042.76, 6158.49 | 6169.23, 35102.36 |
| CT | 0, 550.56 | 555.41, 1307.27 | 1309.63, 2793.18 | 2804.5, 6255.39 | 6310.91, 35862.05 |
| DC | 6.76, 8691.53 | 8738.45, 13191.57 | 13299.9, 20800.83 | 20999.28, 28328.17 | 28603.77, 85747.81 |
| DE | 0, 359.81 | 380.78, 1012.08 | 1032.8, 2078.84 | 2102.9, 4368.04 | 4369.53, 35282.54 |
| FL | 0, 879.51 | 879.76, 2242.52 | 2242.96, 3727.82 | 3729.65, 6143.4 | 6155.48, 177199.45 |
| GA | 0, 131.53 | 131.74, 719.16 | 719.9, 1841.73 | 1841.85, 3035.4 | 3037.54, 45600.25 |
| IA | 0, 19.83 | 19.86, 141.11 | 148.02, 959.12 | 964.13, 3108.19 | 3111.32, 34645.6 |
| ID | 0.33, 29.91 | 31.59, 332.47 | 335.08, 2086.91 | 2132.21, 3980.48 | 4003.76, 23032.53 |
| IL | 0, 469.82 | 473.61, 2139.04 | 2140.68, 4574.31 | 4577.63, 10350.21 | 10377.49, 238333.24 |
| IN | 0, 94.52 | 94.55, 722.32 | 725.54, 1934.78 | 1939.27, 3395.87 | 3397.24, 26698.62 |
| KS | 0, 22.99 | 23.27, 362.09 | 366.6, 2129.86 | 2140.69, 3508.61 | 3514.68, 15547.16 |
| KY | 0, 46.62 | 46.76, 115.33 | 115.66, 818.57 | 820.48, 3158.52 | 3186.92, 13349.69 |
| LA | 0, 73.27 | 73.84, 509.56 | 509.77, 1787.69 | 1798.1, 4059.25 | 4064.27, 22828.01 |
| MA | 0, 720.06 | 720.15, 2004.28 | 2005.29, 5151.07 | 5187.57, 13987.16 | 13987.65, 88458.68 |
| MD | 0, 613.24 | 614.16, 2496.84 | 2501.2, 4505.25 | 4506.56, 7873.38 | 7902.89, 56280.63 |
| ME | 0.47, 46.21 | 46.74, 106.5 | 106.63, 231.54 | 235.59, 846.87 | 857.02, 35931.85 |
| MI | 0, 122.36 | 124.46, 964.77 | 965.98, 2560.07 | 2560.55, 4512.71 | 4513.28, 52377.1 |
| MN | 0, 45.23 | 45.32, 680.13 | 686.23, 2370.5 | 2375.36, 4415.82 | 4423.27, 43837.73 |
| MO | 0, 52.9 | 53.28, 533.76 | 533.92, 1851.96 | 1867.99, 3571.01 | 3571.72, 19922.87 |
| MS | 0, 31.09 | 31.13, 89.22 | 90.05, 523.37 | 529.52, 1351.22 | 1352.28, 7636.77 |
| MT | 0, 3.08 | 3.16, 25.5 | 27.56, 375.2 | 385.88, 2525.41 | 2568.31, 8327.25 |
| NC | 0, 123.52 | 123.86, 412.65 | 413.2, 1191.75 | 1192.72, 2513.7 | 2515.5, 27967.74 |
| ND | 0.63, 3.18 | 3.22, 12.86 | 13.11, 648.44 | 784.9, 3294.6 | 3357.02, 12096.22 |
| NE | 0.29, 13.47 | 13.86, 400.02 | 400.79, 2604.86 | 2622.22, 4287.79 | 4310.27, 14999.01 |
| NH | 0, 95.06 | 95.64, 250.05 | 254.52, 651.41 | 654.75, 2170.29 | 2226.95, 22379.56 |
| NJ | 0, 1352.64 | 1370.11, 3019.21 | 3022.36, 5585.41 | 5599.97, 13703.78 | 13756.71, 122049.49 |
| NM | 0, 22.07 | 22.45, 365.49 | 367.96, 1884.13 | 1893.24, 3891.85 | 3905.24, 15187.62 |
| NV | 0, 1368.55 | 1379.05, 4140.3 | 4141.49, 6523 | 6609.74, 8957.77 | 8981.44, 22474.8 |
| NY | 0, 626.25 | 630.05, 3939.04 | 3948.88, 14689.56 | 14707.15, 46329.35 | 46414.62, 292555.85 |
| OH | 0, 228.29 | 228.54, 1231.68 | 1231.9, 2586.8 | 2593.77, 4571.35 | 4574.64, 30776.1 |
| OK | 0, 41.85 | 42.02, 319.16 | 326.65, 1668.93 | 1695.6, 3515.45 | 3539.13, 11646.94 |
| OR | 0, 101.35 | 102.22, 1464.88 | 1480.78, 3787.82 | 3788.33, 6007.72 | 6008.42, 43660.29 |
| PA | 0, 209.94 | 210.44, 1207.91 | 1215.4, 3190.69 | 3192.6, 7195.8 | 7197.61, 81476.68 |
| RI | 0, 925.63 | 959.96, 2558.36 | 2629.68, 4901.16 | 4904.41, 10170.42 | 10348.91, 24641.02 |
| SC | 0, 83.87 | 83.92, 343.88 | 345.48, 1008.83 | 1010.27, 1964.31 | 2000.55, 14350.85 |
| SD | 0.43, 4.68 | 4.68, 24.52 | 26.47, 313.91 | 317.01, 2310.18 | 2314.36, 7902.38 |
| TN | 0, 81.08 | 81.47, 345.68 | 347.2, 1213.95 | 1218.71, 2606.44 | 2606.79, 41873.68 |
| TX | 0, 331.48 | 333.64, 1895.24 | 1895.32, 3660.49 | 3660.62, 5671.63 | 5671.87, 67990.66 |
| UT | 0, 388.01 | 426.14, 2455.12 | 2474.11, 4318.9 | 4328.37, 6009.73 | 6018.21, 35867.58 |
| VA | 0, 122.93 | 123.85, 1254.08 | 1261.25, 3036.86 | 3040.15, 5314.28 | 5319.05, 102342.62 |
| VT | 0, 33.22 | 33.87, 68.16 | 68.71, 128.46 | 128.7, 747.79 | 752.04, 18626.25 |
| WA | 0, 322.81 | 323.09, 1894.85 | 1900.44, 3788.63 | 3810.66, 6035.36 | 6035.53, 121466.7 |
| WI | 0, 58.79 | 59.36, 530.02 | 531.2, 2091.77 | 2096.95, 4833.08 | 4849.04, 70276.51 |
| WV | 6.25, 35.74 | 36.79, 100.91 | 100.99, 358.03 | 360.77, 1585.57 | 1621.28, 15849.83 |
| WY | 0, 2.55 | 2.56, 80.41 | 81.14, 568.75 | 572.31, 2086.5 | 2091.83, 6987.93 |

**Appendix Table A2.** State Quintile Boundaries for Median Household Income (Min, Max)

| State | Q1 | Q2 | Q3 | Q4 | Q5 |
| --- | --- | --- | --- | --- | --- |
| AK | 26691, 65625 | 66117, 83724 | 83750, 93602 | 94135, 110357 | 111613, 239125 |
| AL | 14962, 41152 | 41237, 53247 | 53460, 65313 | 65315, 82448 | 82500, 240625 |
| AR | 8833, 43036 | 43125, 51870 | 51890, 62077 | 62083, 76203 | 76279, 195880 |
| AZ | 18409, 52618 | 52639, 67321 | 67344, 85280 | 85335, 110707 | 110714, 250001 |
| CA | 5213, 66008 | 66016, 86250 | 86250, 107460 | 107500, 141023 | 141111, 250001 |
| CO | 23590, 65492 | 65568, 83273 | 83317, 103050 | 103140, 132227 | 132538, 250001 |
| CT | 15351, 62146 | 62279, 87857 | 88015, 109197 | 109211, 141250 | 141689, 250001 |
| DC | 12117, 67611 | 68816, 101778 | 102585, 132768 | 132809, 167528 | 167632, 250001 |
| DE | 26611, 61851 | 62864, 77031 | 77036, 89286 | 89720, 113672 | 113949, 203107 |
| FL | 7969, 52303 | 52321, 66202 | 66204, 80000 | 80036, 101853 | 101866, 250001 |
| GA | 8354, 50882 | 50897, 65694 | 65697, 82167 | 82172, 107096 | 107105, 250001 |
| IA | 18520, 58869 | 58971, 68125 | 68155, 77963 | 78000, 91974 | 92083, 198056 |
| ID | 22048, 60781 | 60977, 73290 | 73571, 83333 | 83401, 98642 | 98864, 200652 |
| IL | 2499, 55070 | 55104, 71434 | 71474, 87470 | 87485, 114875 | 114891, 250001 |
| IN | 8606, 49709 | 49709, 63021 | 63029, 76250 | 76292, 90816 | 90882, 250001 |
| KS | 13625, 53071 | 53081, 62535 | 62639, 75964 | 76181, 97188 | 97963, 250001 |
| KY | 9038, 42286 | 42292, 54167 | 54167, 64632 | 64693, 82351 | 82396, 248571 |
| LA | 11473, 37063 | 37148, 51000 | 51023, 65240 | 65313, 84855 | 84898, 222917 |
| MA | 16276, 69675 | 69803, 94692 | 94736, 117629 | 117688, 147596 | 147745, 250001 |
| MD | 2499, 68110 | 68333, 89815 | 89821, 115037 | 115040, 147105 | 147210, 250001 |
| ME | 24353, 56076 | 56089, 66733 | 66890, 78198 | 78229, 94853 | 95066, 195781 |
| MI | 11090, 46759 | 46764, 62731 | 62752, 76538 | 76563, 98224 | 98361, 250001 |
| MN | 16678, 65000 | 65043, 78958 | 78973, 93529 | 93611, 116705 | 116824, 250001 |
| MO | 13849, 48798 | 48804, 60506 | 60507, 73378 | 73438, 91563 | 91711, 250001 |
| MS | 15605, 37422 | 37446, 48182 | 48203, 58958 | 59000, 73061 | 73479, 168750 |
| MT | 26339, 54609 | 54653, 64521 | 64688, 73915 | 74058, 86111 | 86123, 160984 |
| NC | 14821, 49873 | 49896, 61530 | 61533, 74665 | 74688, 95750 | 95767, 250001 |
| ND | 23636, 59688 | 59784, 70927 | 70980, 80208 | 80298, 95083 | 95758, 162000 |
| NE | 24565, 57545 | 57702, 68487 | 68603, 79412 | 79453, 100083 | 100132, 243857 |
| NH | 29625, 73365 | 73625, 89154 | 89238, 105699 | 106000, 123304 | 123581, 207183 |
| NJ | 15943, 68889 | 68929, 91077 | 91111, 113611 | 113921, 149864 | 149895, 250001 |
| NM | 15951, 41545 | 41726, 52549 | 52554, 66136 | 66683, 85944 | 86750, 250001 |
| NV | 18480, 54022 | 54372, 70049 | 70313, 85352 | 85540, 105017 | 105104, 217182 |
| NY | 2499, 55404 | 55441, 74292 | 74297, 94000 | 94000, 124966 | 125000, 250001 |
| OH | 7139, 47384 | 47438, 61357 | 61362, 75813 | 75844, 94928 | 94934, 250001 |
| OK | 11406, 46154 | 46193, 56592 | 56599, 66567 | 66588, 85550 | 85550, 250001 |
| OR | 15653, 60893 | 60921, 74837 | 74845, 89343 | 89375, 109674 | 109848, 250001 |
| PA | 8889, 54024 | 54045, 67417 | 67456, 82135 | 82188, 106250 | 106250, 250001 |
| RI | 16543, 64063 | 64177, 81461 | 82550, 97969 | 98361, 115731 | 116688, 250001 |
| SC | 11833, 45833 | 45878, 58075 | 58077, 70986 | 71000, 87750 | 87877, 250001 |
| SD | 23659, 57546 | 57645, 70000 | 70191, 80000 | 80174, 88007 | 88656, 152958 |
| TN | 2499, 47634 | 47650, 58988 | 59009, 71290 | 71294, 90921 | 91005, 250001 |
| TX | 2499, 49659 | 49674, 64556 | 64583, 81122 | 81125, 108092 | 108097, 250001 |
| UT | 27446, 69214 | 69375, 86250 | 86250, 105154 | 105221, 128013 | 128065, 234808 |
| VA | 11953, 57031 | 57044, 75402 | 75521, 100859 | 100912, 140842 | 141121, 250001 |
| VT | 32212, 66250 | 66273, 76587 | 76597, 85179 | 85368, 97966 | 98365, 164294 |
| WA | 17500, 68790 | 68828, 87925 | 87964, 106187 | 106310, 132625 | 132692, 250001 |
| WI | 10114, 57969 | 58005, 69565 | 69644, 81920 | 81927, 97581 | 97625, 224750 |
| WV | 14943, 43609 | 43732, 52159 | 52237, 61970 | 62000, 73240 | 73689, 135729 |
| WY | 15417, 61346 | 61609, 67823 | 67917, 77213 | 77763, 98782 | 98837, 146829 |

**Appendix Table A3.** State Quintile Boundaries for Percent of Households Receiving SNAP (Min, Max)

| State | Q1 | Q2 | Q3 | Q4 | Q5 |
| --- | --- | --- | --- | --- | --- |
| AK | 0, 0.03 | 0.03, 0.07 | 0.07, 0.1 | 0.1, 0.17 | 0.17, 0.6 |
| AL | 0, 0.05 | 0.05, 0.1 | 0.1, 0.16 | 0.16, 0.24 | 0.24, 0.9 |
| AR | 0, 0.04 | 0.04, 0.07 | 0.07, 0.11 | 0.11, 0.17 | 0.17, 0.64 |
| AZ | 0, 0.02 | 0.02, 0.06 | 0.06, 0.11 | 0.11, 0.19 | 0.19, 0.71 |
| CA | 0, 0.04 | 0.04, 0.08 | 0.08, 0.13 | 0.13, 0.21 | 0.21, 1 |
| CO | 0, 0.02 | 0.02, 0.05 | 0.05, 0.09 | 0.09, 0.14 | 0.14, 0.54 |
| CT | 0, 0.02 | 0.02, 0.05 | 0.05, 0.1 | 0.1, 0.22 | 0.22, 1 |
| DC | 0, 0.02 | 0.02, 0.05 | 0.05, 0.12 | 0.12, 0.29 | 0.3, 0.69 |
| DE | 0, 0.03 | 0.03, 0.07 | 0.08, 0.11 | 0.11, 0.18 | 0.18, 0.52 |
| FL | 0, 0.03 | 0.03, 0.07 | 0.07, 0.13 | 0.13, 0.22 | 0.22, 0.93 |
| GA | 0, 0.03 | 0.03, 0.08 | 0.08, 0.13 | 0.13, 0.22 | 0.22, 1 |
| IA | 0, 0.04 | 0.04, 0.06 | 0.06, 0.09 | 0.09, 0.14 | 0.14, 0.45 |
| ID | 0, 0.02 | 0.02, 0.05 | 0.05, 0.08 | 0.08, 0.11 | 0.11, 0.62 |
| IL | 0, 0.05 | 0.05, 0.09 | 0.09, 0.15 | 0.15, 0.25 | 0.25, 0.8 |
| IN | 0, 0.03 | 0.03, 0.05 | 0.05, 0.09 | 0.09, 0.17 | 0.17, 0.73 |
| KS | 0, 0.02 | 0.02, 0.04 | 0.04, 0.07 | 0.07, 0.12 | 0.12, 1 |
| KY | 0, 0.05 | 0.05, 0.1 | 0.1, 0.15 | 0.15, 0.22 | 0.22, 0.63 |
| LA | 0, 0.08 | 0.08, 0.13 | 0.13, 0.2 | 0.2, 0.28 | 0.28, 0.87 |
| MA | 0, 0.04 | 0.04, 0.08 | 0.08, 0.13 | 0.13, 0.24 | 0.24, 1 |
| MD | 0, 0.03 | 0.03, 0.07 | 0.07, 0.11 | 0.11, 0.18 | 0.18, 0.62 |
| ME | 0, 0.05 | 0.05, 0.08 | 0.08, 0.13 | 0.13, 0.19 | 0.19, 0.65 |
| MI | 0, 0.04 | 0.04, 0.08 | 0.08, 0.13 | 0.13, 0.24 | 0.24, 0.82 |
| MN | 0, 0.02 | 0.02, 0.05 | 0.05, 0.07 | 0.07, 0.12 | 0.12, 0.67 |
| MO | 0, 0.03 | 0.03, 0.06 | 0.06, 0.1 | 0.1, 0.17 | 0.17, 0.68 |
| MS | 0, 0.05 | 0.05, 0.1 | 0.1, 0.15 | 0.15, 0.24 | 0.24, 0.6 |
| MT | 0, 0.03 | 0.03, 0.05 | 0.05, 0.08 | 0.08, 0.13 | 0.13, 0.46 |
| NC | 0, 0.04 | 0.04, 0.09 | 0.09, 0.14 | 0.14, 0.21 | 0.21, 0.84 |
| ND | 0, 0.02 | 0.02, 0.04 | 0.04, 0.06 | 0.06, 0.1 | 0.1, 0.39 |
| NE | 0, 0.03 | 0.03, 0.05 | 0.05, 0.08 | 0.08, 0.13 | 0.13, 0.46 |
| NH | 0, 0.01 | 0.01, 0.03 | 0.03, 0.06 | 0.06, 0.1 | 0.1, 0.4 |
| NJ | 0, 0.01 | 0.01, 0.04 | 0.04, 0.08 | 0.08, 0.16 | 0.16, 1 |
| NM | 0, 0.08 | 0.09, 0.15 | 0.15, 0.22 | 0.22, 0.32 | 0.32, 0.91 |
| NV | 0, 0.04 | 0.04, 0.08 | 0.08, 0.13 | 0.13, 0.21 | 0.21, 0.55 |
| NY | 0, 0.04 | 0.04, 0.08 | 0.08, 0.15 | 0.15, 0.26 | 0.26, 0.87 |
| OH | 0, 0.04 | 0.04, 0.08 | 0.08, 0.13 | 0.13, 0.23 | 0.23, 1 |
| OK | 0, 0.06 | 0.06, 0.1 | 0.1, 0.15 | 0.15, 0.22 | 0.22, 1 |
| OR | 0, 0.08 | 0.08, 0.12 | 0.12, 0.17 | 0.17, 0.24 | 0.24, 0.56 |
| PA | 0, 0.04 | 0.04, 0.09 | 0.09, 0.14 | 0.14, 0.24 | 0.24, 1 |
| RI | 0, 0.04 | 0.04, 0.07 | 0.07, 0.12 | 0.12, 0.24 | 0.26, 0.62 |
| SC | 0, 0.03 | 0.03, 0.08 | 0.08, 0.12 | 0.12, 0.2 | 0.2, 0.73 |
| SD | 0, 0.02 | 0.02, 0.05 | 0.05, 0.08 | 0.08, 0.13 | 0.13, 0.67 |
| TN | 0, 0.03 | 0.03, 0.07 | 0.07, 0.12 | 0.12, 0.19 | 0.19, 0.97 |
| TX | 0, 0.03 | 0.03, 0.07 | 0.07, 0.12 | 0.12, 0.21 | 0.21, 0.71 |
| UT | 0, 0.01 | 0.01, 0.03 | 0.03, 0.05 | 0.05, 0.08 | 0.08, 0.32 |
| VA | 0, 0.02 | 0.02, 0.05 | 0.05, 0.09 | 0.09, 0.16 | 0.16, 0.76 |
| VT | 0.01, 0.06 | 0.06, 0.08 | 0.08, 0.11 | 0.11, 0.15 | 0.15, 0.3 |
| WA | 0, 0.04 | 0.04, 0.07 | 0.07, 0.12 | 0.12, 0.19 | 0.19, 0.56 |
| WI | 0, 0.04 | 0.04, 0.07 | 0.07, 0.11 | 0.11, 0.18 | 0.18, 0.78 |
| WV | 0.01, 0.1 | 0.1, 0.14 | 0.14, 0.19 | 0.19, 0.27 | 0.27, 0.57 |
| WY | 0, 0.01 | 0.01, 0.04 | 0.04, 0.05 | 0.05, 0.09 | 0.09, 0.21 |

**Appendix Table A4.** Waiver characteristics and analytic sample by state

| **State** | **Waiver type** | **Submitted** | **Implemented** | **Treated tracts** | **Convenience tracts,**  **No. (%)** | **Supermarket/ superstore tracts, No. (%)** |
| --- | --- | --- | --- | --- | --- | --- |
| IA | Beverages + candy + foods | Apr 1, 2025 | Jan 1, 2026 | 893 | 715 (80.1) | 316 (35.4) |
| IN | Beverages and candy | Apr 15, 2025 | Jan 1, 2026 | 1,684 | 1,245 (73.9) | 489 (29.0) |
| NE | Beverages only | Apr 14, 2025 | Jan 1, 2026 | 551 | 331 (60.1) | 175 (31.8) |
| UT | Beverages only | May 16, 2025 | Jan 1, 2026 | 710 | 411 (57.9) | 242 (34.1) |
| WV | Beverages only | Jun 10, 2025 | Jan 1, 2026 | 545 | 453 (83.1) | 176 (32.3) |
| ID | Beverages and candy | May 16, 2025 | Feb 15, 2026 | 456 | 298 (65.4) | 146 (32.0) |
| OK | Beverages + candy | Jun 10, 2025 | Feb 15, 2026 | 1,195 | 881 (73.7) | 349 (29.2) |
| LA | Beverages + candy | Jun 27, 2025 | Feb 18, 2026 | 1,365 | 899 (65.9) | 433 (31.7) |
| TX | Beverages + candy | Jun 26, 2025 | Apr 1, 2026 | 6,831 | 5,077 (74.3) | 2,173 (31.8) |
| FL | Beverages + candy + foods | May 29, 2025 | Apr 20, 2026 | 5,074 | 3,226 (63.6) | 1,991 (39.2) |
| Total |  |  |  | 19,304 | 13,536 (70.1) | 6,490 (33.6) |

Note: Analytic sample columns report the number of census tracts in treated states with at least one store of the relevant type in any biweekly period before the first waiver submission observed in the sample. Waiver types describe the categories of products covered by the implemented SNAP waiver restrictions.

**Appendix Table A5.** SNAP caseload trends and sensitivity of retailer estimates to contemporaneous SNAP participation

| **Panel A. Differential changes in state SNAP caseloads after H.R.1** | | | | |
| --- | --- | --- | --- | --- |
| **Caseload measure** | **Differential change in waiver states, %** | **95% CI** | **P- value** | **State-month observations** |
| SNAP households | −2.06 | −4.83 to 0.80 | 0.152 | 800 |
| SNAP persons | −2.08 | −4.99 to 0.91 | 0.166 | 800 |
|  |  |  |  |  |
| **Panel B. SNAP-authorized convenience stores** | | | | |
| **Model** | **Estimated change, %** | **Change in stores per 100 tracts (95% CI)** | **P-value** | **Tract-period observations** |
| Primary model, full study period | −1.43 | −3.05 (−5.72 to −0.37) | 0.026 | 1,849,914 |
| Primary model, months with caseload data | −1.21 | −2.58 (−5.26 to 0.11) | 0.060 | 1,737,798 |
| + contemporaneous SNAP households | −1.22 | −2.60 (−5.35 to 0.16) | 0.064 | 1,737,798 |
| + contemporaneous SNAP persons | −1.18 | −2.52 (−5.30 to 0.26) | 0.075 | 1,737,798 |
|  |  |  |  |  |
| **Panel C. SNAP-authorized supermarkets and superstores** | | | | |
| **Model** | **Estimated change, %** | **Change in stores per 100 tracts (95% CI)** | **P-value** | **Tract-period observations** |
| Primary model, full study period | −0.28 | −0.38 (−1.56 to 0.80) | 0.522 | 913,110 |
| Primary model, months with caseload data | −0.28 | −0.38 (−1.65 to 0.88) | 0.545 | 857,770 |
| + contemporaneous SNAP households | −0.40 | −0.54 (−1.78 to 0.70) | 0.388 | 857,770 |
| + contemporaneous SNAP persons | −0.37 | −0.50 (−1.75 to 0.75) | 0.423 | 857,770 |

Note: Panel A reports the waiver-state × post-H.R.1 coefficient from state-month regressions of log SNAP caseloads with state and month fixed effects and standard errors clustered by state. Coefficients and confidence intervals are transformed to percentage changes as 100 × [exp(coefficient) − 1]. H.R.1 was enacted in July 2025. Panels B and C report the coefficient on waiver implementation from the adjusted two-way fixed-effects difference-in-differences models. The full-study-period model uses all available retailer observations through June 23, 2026. The remaining models are restricted to months with contemporaneous USDA SNAP caseload data, which were available through May 2026. Contemporaneous SNAP household and person counts are entered in logarithmic form. Retailer models include the post-submission/pre-implementation indicator, state and biweekly-period fixed effects, log population density, SNAP household share, White population share, median household income, and the post-H.R.1 × baseline SNAP participation interaction. Standard errors are clustered by state. Percentage retailer changes are calculated relative to the treated-state pre-implementation mean.

**Appendix Table A6.** Conventional and wild-cluster-bootstrap inference for primary waiver implementation estimates

| Outcome | Estimate, stores per 100 tracts | Conventional 95% CI | Conventional P-value | Wild-bootstrap  95% CI | Wild-bootstrap  P-value |
| --- | --- | --- | --- | --- | --- |
| Convenience stores | −3.05 | −5.72 to −0.37 | 0.026 | −5.80 to 0.29 | 0.075 |
| Supermarkets/superstores | −0.38 | −1.56 to 0.80 | 0.522 | −1.96 to 0.92 | 0.549 |

Note: Estimates are coefficients on the waiver implementation indicator from the primary adjusted two-way fixed-effects difference-in-differences models and are expressed as changes in SNAP-authorized stores per 100 census tracts. Conventional 95% confidence intervals and P-values use standard errors clustered by state. Wild-cluster-bootstrap inference also clusters by state and uses 9,999 replications with Webb weights and symmetric two-sided P-values. For the bootstrap analysis, the primary specification was estimated equivalently by absorbing state fixed effects and including biweekly-period fixed effects as indicator variables. Both specifications also include the post-submission/pre-implementation indicator and the covariates used in the primary analysis.
